# Assessing abnormal corneal endothelial cells from in vivo confocal microscopy images using a fully automated deep learning system

**DOI:** 10.1186/s40662-023-00340-7

**Published:** 2023-06-01

**Authors:** Jinghao Qu, Xiaoran Qin, Rongmei Peng, Gege Xiao, Shaofeng Gu, Haikun Wang, Jing Hong

**Affiliations:** 1 Department of Ophthalmology, Peking University Third Hospital, No.49 Garden North Road, Haidian, Beijing, 100191 China; 2 Beijing Key Laboratory of Restoration of Damaged Ocular Nerve, Peking University Third Hospital, Beijing, China; 3 Research Center for Brain-inspired Intelligence, Institute of Automation, Chinese Academy of Sciences, Beijing, China

**Keywords:** Abnormal corneal endothelial cells, LASER in vivo confocal microscopy, Deep learning

## Abstract

**Background:**

The goal of this study is to develop a fully automated segmentation and morphometric parameter estimation system for assessing abnormal corneal endothelial cells (CECs) from LASER in vivo confocal microscopy (IVCM) images.

**Methods:**

First, we developed a fully automated deep learning system for assessing abnormal CECs using a previous development set composed of normal images and a newly constructed development set composed of abnormal images. Second, two testing sets, one with 169 normal images and the other with 211 abnormal images, were used to evaluate the clinical validity and effectiveness of the proposed system on LASER IVCM images with different corneal endothelial conditions, particularly on abnormal images. Third, the automatically calculated endothelial cell density (ECD) and the manually calculated ECD were compared using both the previous and proposed systems.

**Results:**

The automated morphometric parameter estimations of the average number of cells, ECD, coefficient of variation in cell area and percentage of hexagonal cells were 257 cells, 2648 ± 511 cells/mm^2^, 32.18 ± 6.70% and 56.23 ± 8.69% for the normal CEC testing set and 83 cells, 1450 ± 656 cells/mm^2^, 34.87 ± 10.53% and 42.55 ± 20.64% for the abnormal CEC testing set. Furthermore, for the abnormal CEC testing set, Pearson’s correlation coefficient between the automatically and manually calculated ECDs was 0.9447; the 95% limits of agreement between the manually and automatically calculated ECDs were between 329.0 and − 579.5 (concordance correlation coefficient = 0.93).

**Conclusions:**

This is the first report to count and analyze the morphology of abnormal CECs in LASER IVCM images using deep learning. Deep learning produces highly objective evaluation indicators for LASER IVCM corneal endothelium images and greatly expands the range of applications for LASER IVCM.

## Background

Normal corneal endothelial cells (CECs) form regular endothelial mosaics of hexagonal cells of approximately the same size with well-defined cell boundaries. The corneal endothelial cell density (ECD) decreases (that is, the mean cell area increases) with age [1]; the most rapid loss occurs from birth to the first few years of life [2], then stabilizes from 20 years of age through approximately 50 years [3], and significantly decreases again after the age of 60 years [4]. On average, age-related cell loss is approximately 0.5% per year [5]. A higher variability in polymegethism and pleomorphism has also been shown to correlate with age [6]. Moreover, the endothelial cell morphology among patients with acute angle closure or chronic open-angle glaucoma, uveitis, keratitis, and eye surgeries (cataract phacoemulsification or corneal endothelial transplantation) may become abnormal. Morphological recognition of abnormal CECs is of great importance in the clinic but makes identifying cell boundaries more difficult.

The three parameters commonly used to address such irregularities are the ECD, percentage of hexagonal cells (HEX), and coefficient of variation in cell area (CV). The most common method for measuring these parameters in the clinic is specular microscopy. However, this technique requires a regular, smooth endothelial surface for acquiring high-quality images; although this is the norm in healthy corneas, an abnormal corneal endothelial layer will have fewer CECs, a reduced HEX and cells with abnormal intensity patterns, leading to multiple inconsistent manual analyses of the images for properly assessing the endothelial layer. When the CECs are moderately abnormal (endothelium was normal), specular images of these cells are prone to low contrast, high noise levels, and blurred cell boundaries. Moreover, many recent studies have shown a lack of agreement among different automatic built-in specular microscopy software programs and sometimes an overestimation of the ECD with automatic segmentation relative to manual segmentation, suggesting that the automatic results should be used with caution [7–9].

LASER in vivo confocal microscopy (IVCM) is a high-resolution, high-speed, digital confocal laser scanning microscope technique that permits in vivo investigations of the cornea. Due to its high resolution, LASER IVCM can be used to identify abnormal CECs. However, the Heidelberg Eye Explorer [10] of the Heidelberg Retina Tomograph (HRT) III requires manual operation, which can be time consuming, highly subjective, and tedious and does not allow the geometric analysis of endothelial cell shape. Unlike the ECD, neither the HEX nor the CV can be obtained from the Heidelberg Eye Explorer. These two parameters are very important indicators for evaluating abnormal CEC function for guiding diagnosis and treatment. According to a literature review, there are no fully automated segmentation and quantification systems for identifying (i.e., calculating the ECD, HEX and CV of) abnormal CECs in LASER IVCM images.

Existing fully automated segmentation and quantification systems for abnormal CECs mainly involve the use of specular microscopic images [11, 12]. In those studies, post-corneal transplantation specular microscopy images were used to train a convolutional neural network (CNN). However, the corneal endothelial condition described in those two studies was found to be relatively mild, and certain severe endothelial abnormalities were not included.

In a previous study, our team developed a fully automated segmentation and quantification system for normal LASER IVCM images (hereafter referred to as *system_normal*) [13]. On LASER IVCM images, abnormal CECs present with low density and morphological abnormalities, resulting in substantial challenges for identification. Therefore, in this study, abnormal LASER IVCM corneal endothelial images were used to develop an automated system that assesses abnormal CECs. The comparison with *system_normal* was also conducted in this study. This is the first attempt using deep learning to calculate the ECD, HEX and CV of abnormal CECs from LASER IVCM images.

## Materials and methods

### Image capture

In this prospective study, images of CECs were acquired using a LASER IVCM system (HRT III Rostock Cornea Module [RCM]; Heidelberg Engineering GmbH, Heidelberg, Germany). The specific steps of LASER IVCM image acquisition have been described in a previous article [13]. Scanning depth was increased to approximately 450–600 μm when the CECs could be seen clearly. The images were taken from the central cornea using section mode (a single image was acquired and stored each time the footswitch or the acquisition button was pressed) and saved in JPG format with 8-bit gray levels and a size of 384 × 384 pixels (400 × 400 μm). Three-good quality images (image quality > 80) of the CECs were selected for cell density counting. The same technician selected a clear region of interest (ROI) from the LASER IVCM image and placed a mark on each CEC. The ECD was manually calculated based on the number of cells within the given ROI (larger than 50% of the image) in the daytime. The average of three measurements was used for further analysis. Manual ECD measurements were performed using the Heidelberg Eye Explorer. The study was performed according to the tenets of the Declaration of Helsinki and was approved by the institutional review board of Peking University Third Hospital (IRB00006761-M2022834).

### Study subject

Both the normal and abnormal groups were diagnosed by an experienced ophthalmologist (JH) at Peking University Third Hospital from April 2020 to January 2022. For the normal group: potential subjects were excluded from the study if they had undergone previous corneal or ocular surgery, had any ocular pathology and keratopathy, or had chronic use of topical ocular medications. For the abnormal group: patients with acute angle closure or chronic open-angle glaucoma, uveitis, keratitis, and eye surgeries (cataract phacoemulsification or corneal endothelial transplantation) were included in this study; potential subjects were excluded from the study if the CEC images could not be counted. One hundred and seventy-six patients were included as the abnormal group (randomly split into development and testing set) and 24 patients were excluded.

### Datasets

**Fig. 1:**
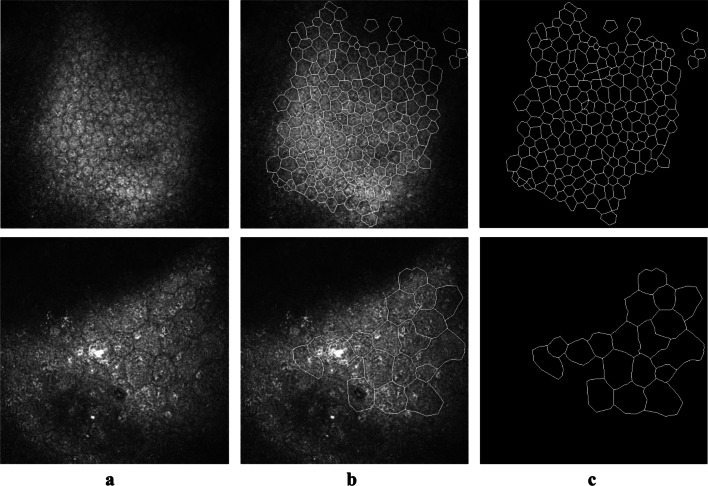
Two examples of the LASER in vivo confocal microscopy (IVCM) image annotations. The first and second rows indicate a normal and an abnormal LASER IVCM image, respectively. **a** Original LASER IVCM image; **b** Annotated image with manually traced cell contours; **c** Binary ground-truth image

To develop the automated system, a development set containing 231 LASER IVCM images from 156 patients was used. The 231 LASER IVCM images consisted of 99 normal LASER IVCM images (99 eyes of 99 patients) from a previous work [13] and 132 abnormal LASER IVCM images from 57 eyes of 57 patients. Two independent resident ophthalmologists manually labeled the cell contours using an open-source image manipulation program (GIMP) to generate ground-truth results from which manual morphometric parameters could be determined. Two examples of the LASER IVCM image annotations and the binary ground-truth results are shown in Fig. 1. Constructing the development set from both normal and abnormal LASER IVCM images is helpful for building a robust system that can be applied to various clinical scenarios. Specifically, the manual morphometric parameters for the 99 normal LASER IVCM images were an average number of cells of 143, an average ECD of 2456 cells/mm^2^, an average CV of 36% and an average HEX of 52%. The corresponding parameters for the 132 abnormal LASER IVCM images were 28 cells, 711 cells/mm^2^, 42% and 23%, respectively. This shows that the abnormal LASER IVCM images had fewer cells, a lower cell density, a higher variation in cell size and more nonhexagonal cells.

**Fig. 2:**
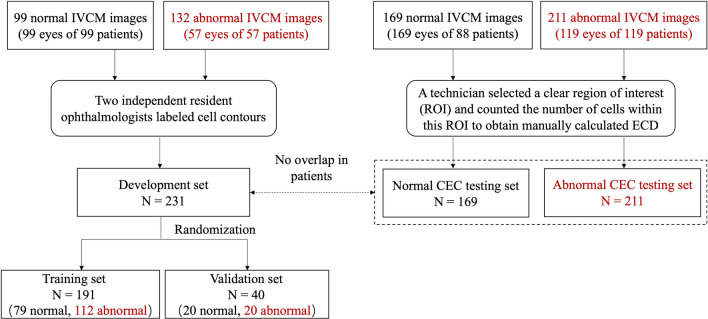
Dataset construction flowchart. The newly added parts for the proposed system (different from system_normal) are marked in red. IVCM, in vivo confocal microscopy; CEC, corneal endothelial cell; ECD, endothelial cell density

The proposed system was tested on two other testing datasets, namely, the normal CEC testing set and the abnormal CEC testing set. We used the testing set from a previous work [13] but excluded nine patients diagnosed with Fuchs endothelial dystrophy or corneal endotheliitis, thereby leaving 169 LASER IVCM images from 169 eyes of 88 subjects (36 males and 52 females; average age 65.6 ± 12.6 years) as the normal CEC testing set. For the abnormal CEC testing set, we newly collected 211 LASER IVCM images from 119 eyes of 119 patients (60 males and 59 females), age ranging from 12 to 90 years (average age 61.1 ± 16.6 years). The diagnoses of the abnormal CEC patients included acute angle-closure glaucoma, cataracts after phacoemulsification and post-Descemet’s stripping automated endothelial keratoplasty (post-DSAEK). On LASER IVCM images, abnormal CECs present with low density and/or morphological abnormalities. It should be noted that the patients included in the two testing sets did not overlap with the patients in the development set. The ECDs of all testing images were manually calculated by the same technician who selected a clear ROI and counted the number of cells within this ROI. The flowchart for dataset construction is shown in Fig. 2.

### Development of the automated algorithm

**Fig. 3:**
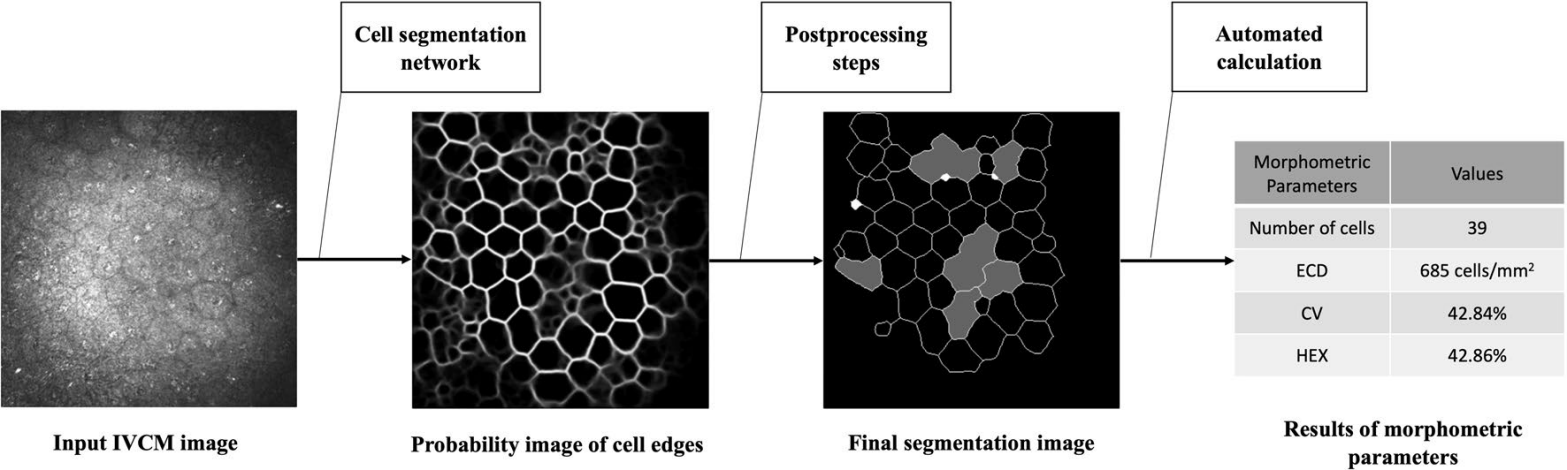
Pipeline of the fully automated segmentation and morphometric parameter estimation system. In the final segmentation image, abnormally segmented cells are indicated (abnormally small cells are marked in white, and abnormal, nonconvex cells are marked in dark gray) and excluded from the automated calculation of the morphometric parameters. IVCM, in vivo confocal microscopy; ECD, endothelial cell density; CV, coefficient of variation in cell area; HEX, percentage of hexagonal cells

For the input LASER IVCM images, a cell segmentation network was established to generate probability images of the cell edges, which were then subjected to a series of postprocessing steps to produce the final segmentation images, from which the automated morphometric parameters could be calculated. The entire pipeline of the proposed system is presented in Fig. 3.

To train the cell segmentation network, the development set was randomly divided into a training set (191 images, used to train the network to identify cell edges) and a validation set (40 images, used to evaluate the network every few training epochs), as shown in Fig. 2. To help the network learn the characteristics of cell edges in a stable manner, one-pixel-width cell boundaries in ground-truth images were thickened by a morphological dilation operation to create target images. Then, all input LASER IVCM images were normalized to values ranging from 0 to 1 due to contrast inhomogeneity. During training, data augmentation techniques were conducted on the training set, including random scaling, cropping and flipping. Based on previous work [12] where all training images were randomly scaled from 1.0 to 2.0, in this study, we narrowed the scale range to 1.0 to 1.2 for newly added abnormal LASER IVCM images that already had a rather low density. The cell segmentation network was a fully convolutional network with an encoder-decoder architecture, where the encoder part extracts relevant features and the decoder fuses multiscale features to output a probability image of cell edges. We completely trained this network over 500 epochs. The details of the network architecture and other training configuration settings were presented in our previous work [13].

After obtaining the cell edge probability images, a series of postprocessing steps were conducted to produce the final segmentation images [12]. Briefly, the probability images were binarized using the adaptive Otsu algorithm and cell boundaries at the image borders were then discarded. Morphological thinning and pruning operations were performed sequentially to generate one-pixel-width enclosed boundary images. Subsequently, abnormally segmented cells were removed according to predefined criteria and the experience of the blinded ophthalmologists. This produced the final segmentation images from which morphometric parameters including the number of cells, ECD, CV and HEX, could be calculated automatically.

### Statistical analysis

Statistical analysis was performed with SPSS 18.0 (SPSS, Inc., Chicago, IL, USA). For the two testing sets, the correlation between the automatically and manually calculated ECDs was examined using the Pearson test. The ECDs estimated by the proposed system and *system_normal* with the abnormal testing set were compared with the paired-samples t test. All tests were two-tailed with *P <* 0.05 was considered statistically significant. Bland-Altman analysis was used to evaluate the agreement between the automatically and manually calculated ECDs. The area under the receiver operating characteristic curve (AUC), sensitivity and specificity were calculated based on the probability output images and the binary target images to evaluate the performance of the proposed cell segmentation network. The relative error between the automatically calculated morphometric parameters and the corresponding manually calculated values was also calculated to evaluate the reliability of our morphometric parameter estimation system.

## Results

### Results with the validation set

Although the validation set (40 images) was generated randomly from images from the development set, we ensured that it included 20 normal LASER IVCM images and 20 abnormal LASER IVCM images. The proposed cell segmentation network achieved an AUC of 0.9436, sensitivity of 0.6483 and specificity of 0.9504. The average relative errors of the number of cells, ECD, CV and HEX between the automated values and manual values were 16.46%, 13.14%, 17.99% and 29.68%, respectively. When analyzing the normal and abnormal LASER IVCM images separately, we found that based on manual calculation, the average number of cells for the 20 normal images and 20 abnormal images in the validation set was 139 cells and 37 cells, respectively, and the average ECDs were 2602 cells/mm^2^ and 821 cells/mm^2^, respectively. The average relative errors of the number of cells, ECD, CV and HEX for the 20 normal images were 16.44%, 6.92%, 13.93% and 13.48%, respectively, while the corresponding values for the 20 abnormal images were 16.48%, 19.36%, 22.04% and 45.88%, respectively. The above results show that (1) the abnormal images had a much lower number of cells and lower cell density than the normal images, indicating that the abnormal corneal endothelial condition in our study was very severe; (2) it was more challenging to accurately estimate the morphometric parameters for the abnormal images; and (3) when the segmentation results were inconsistent with the manual labels, the estimations were more easily affected for images with a lower number of cells, especially the estimations of HEX.

### Results with the two testing sets

**Fig. 4:**
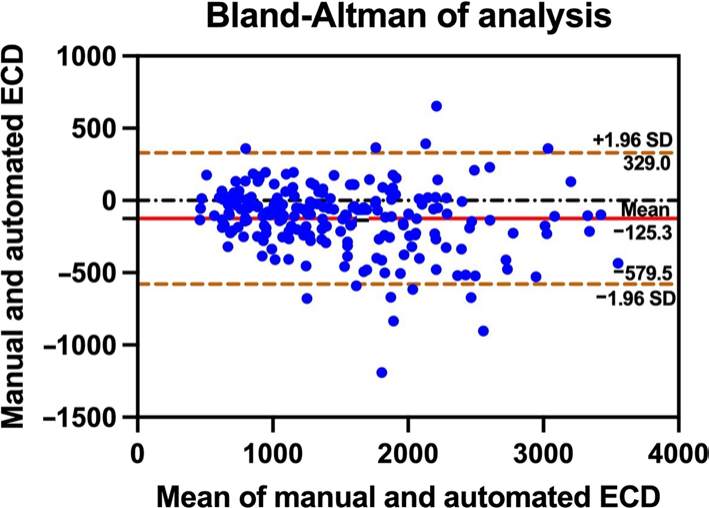
Bland-Altman plot comparing the manually and automatically calculated ECDs for the abnormal CEC testing set using the proposed system. CEC, corneal endothelial cell; ECD, endothelial cell density

**Fig. 5:**
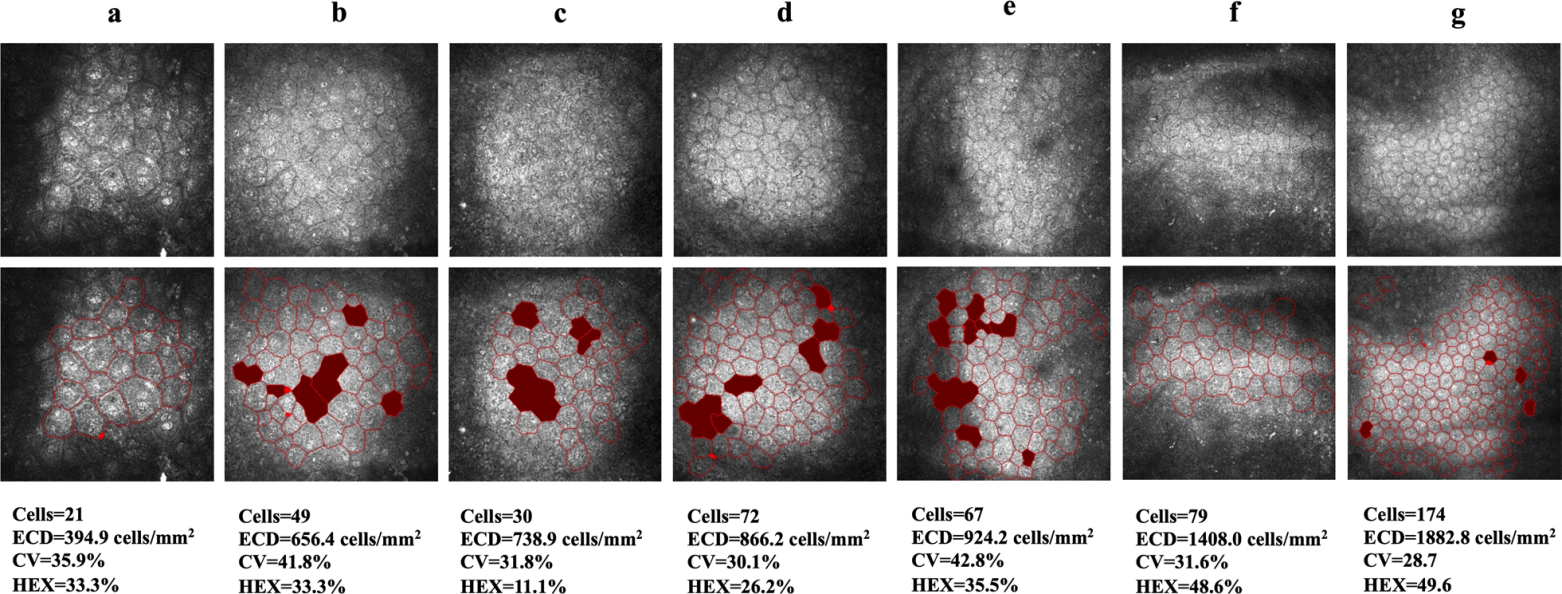
Seven examples of abnormal CEC images from patients with different severities were recognized by the proposed system. The cells in red indicate abnormally segmented cells that were excluded from the calculations. **a** ECD under 500 cells/mm^2^; **b** to **e** ECD under 1000 cells/mm^2^; **f** ECD under 1500 cells/mm^2^; **g** ECD under 2000 cells/mm^2^. The automated estimations are shown below. CEC, corneal endothelial cell; ECD, endothelial cell density; CV, coefficient of variation in cell area; HEX, percentage of hexagonal cells

For all images in the normal and abnormal CEC testing sets, only the manually calculated ECDs were available for comparison. The Pearson’s correlation coefficient between the automatically and manually calculated ECD was 0.8470 for the normal CEC testing set ({manually calculated ECD} = 225.837 + 0.892{the proposed system}, *P* = 0.055 > 0.05) and 0.9447 for the abnormal CEC testing set ({manually calculated ECD} = 104.991 + 1.014{the proposed system}, *P* = 0.000 < 0.05*).* The 95% limits of agreement between the manually and automatically calculated ECD were between 329.0 and − 579.5 (concordance correlation coefficient = 0.93, Fig. 4) for the abnormal CEC testing set using the proposed system. Examples of abnormal CEC images from patients with different severities are presented in Fig. 5.

The inclusion of abnormal LASER IVCM images in the development set will be very helpful for expanding the application scope of our system in clinical trials, where such abnormal cases more urgently require an accurate diagnosis. To demonstrate this point, we also evaluated *system_normal* on the two testing sets for comparison. The Pearson’s correlation coefficient between the ECD calculated by *system_normal* and the manually calculated ECD was 0.8491 for the normal CEC testing set ({manually calculated ECD} = 212.203 + 0.898{*system_normal*}, *P* = 0.07 > 0.05) and 0.9220 for the abnormal CEC testing set ({manually calculated ECD} = 4.636 + 1.047{*system_normal*}, *P* = 0.925 > 0.05). The 95% limits of agreement between the manually and automatically calculated ECDs were between 614.6 and − 463.1 (concordance correlation coefficient = 0.91) for the abnormal CEC testing set. Comparing the above results of *system_normal* with those of the proposed system in this study, we can see that the proposed system (1) yields a substantial 0.0227 improvement in the correlation between the ECDs for abnormal images but a slight 0.0021 reduction in the correlation between the ECDs for normal images; (2) yields a 0.02 improvement in the concordance correlation coefficient between the ECDs for abnormal images; and (3) is more effective in estimating cell density for both normal and abnormal images.

**Fig. 6:**
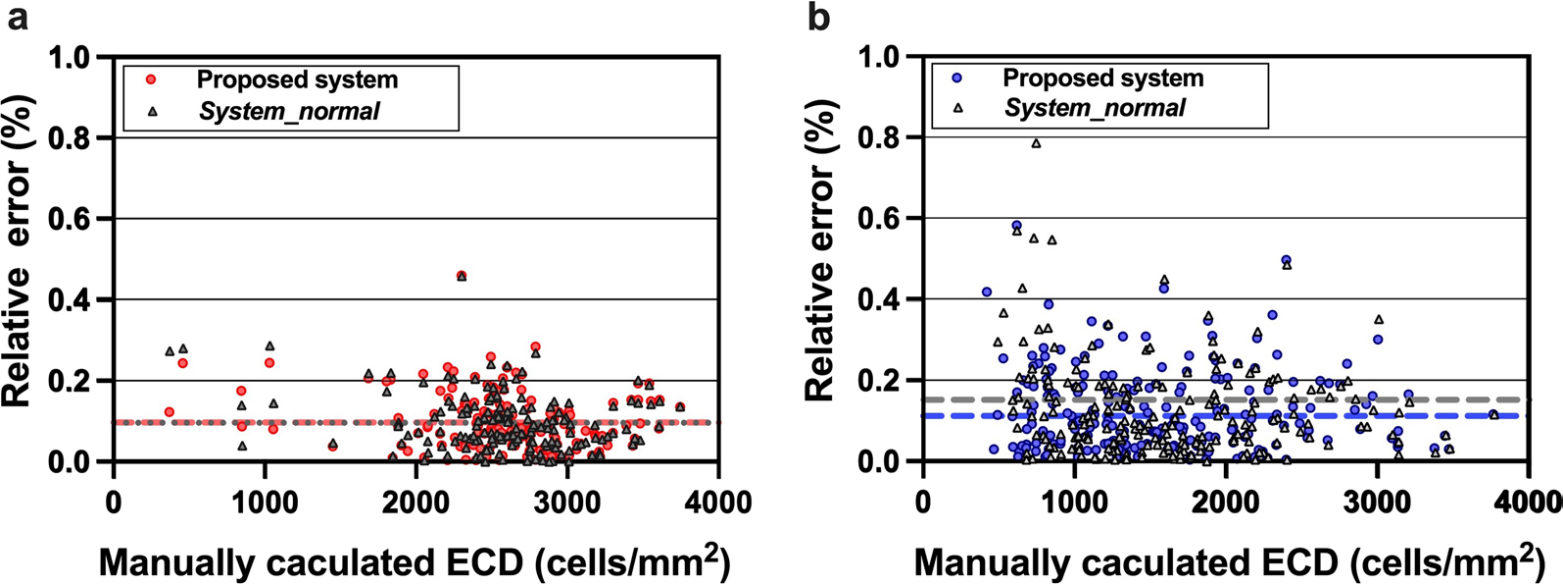
Relative error of the estimates of ECD using the proposed system (colored circle) and *system_normal* (gray triangle) for the normal and abnormal CEC testing sets. **a** Relative error of the ECD using the two systems for the normal CEC testing set (the average relative errors were almost the same); **b** relative error of the ECD using the two systems for the abnormal CEC testing set. The dotted lines indicate the average relative errors. CEC, corneal endothelial cell; ECD, endothelial cell density

Using the proposed system, the average relative error between the automatically and manually calculated ECDs was 0.0957 for the normal CEC testing set and 0.1245 for the abnormal CEC testing set; in comparison, the average relative error between the ECD calculated from *system_normal* and the manually calculated ECD was 0.0966 for the normal CEC testing set and 0.1522 for the abnormal CEC testing set. This also shows the effectiveness of the proposed system in estimating cell density in LASER IVCM images with widely varying ECDs. In each image, the relative error tended to decrease as the ECD increased (Fig. 6).

**Fig. 7:**
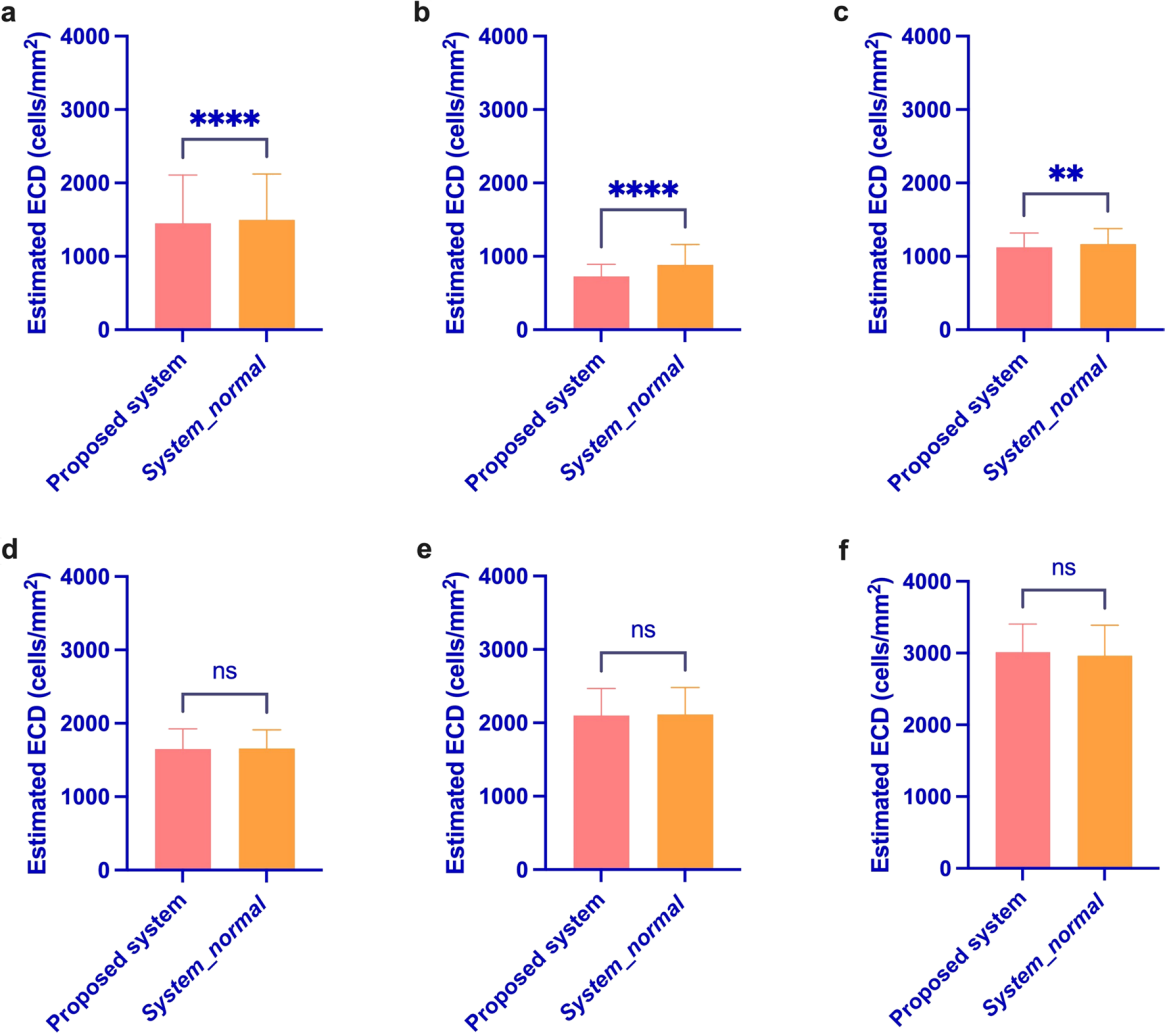
Results of the paired-samples t test using the two systems with the abnormal testing set (****P < 0.001, **P < 0.01, ns = not significant). **a** Manually calculated ECD from 0–4000 cells/mm^2^. **b** Manually calculated ECD from 0–999 cells/mm^2^. **c** Manually calculated ECD from 1000–1499 cells/mm^2^. **d** The manually calculated ECD from 1500–1999 cells/mm^2^. **e** Manually calculated ECD from 2000–2999 cells/mm^2^. **f** Manually calculated ECD from 3000–4000 cells/mm^2^. ECD, endothelial cell density

**Fig. 8:**
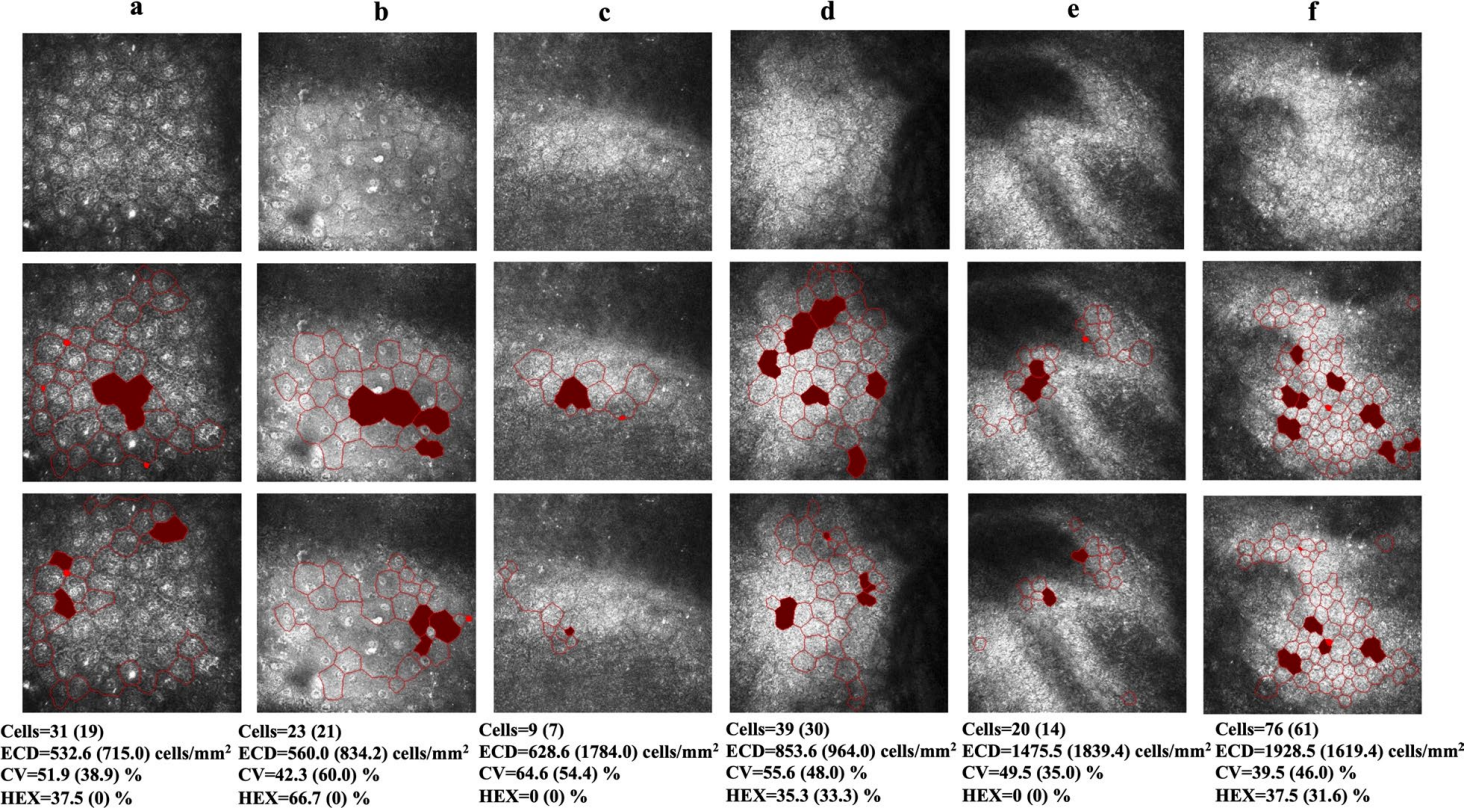
Six examples of abnormal images (first row) recognized by the proposed system (second row) and *system_normal* (third row). **a** to **f** were abnormal images; **a** to **d** ECD under 1000 cells/mm^2^; **e**, **f** ECD under 2000 cells/mm^2^. The cells in red indicate abnormally segmented cells that were excluded from the calculations. The automated estimations are indicated below the corresponding images (estimations by *system_normal* are given in parenthesis). CEC, corneal endothelial cell; ECD, endothelial cell density; HEX, percentage of hexagonal cells

To further contrast the two systems, the ECDs estimated by the proposed system and *system_normal* for the abnormal testing set were compared using the paired-samples t test, which revealed a significant difference between the two systems (t = − 4.709, *P* = 0.000 < 0.001, Fig. 7a). When the manually calculated ECD was under 999 and 1000–1499 cells/mm^2^, there was a significant difference between the two systems (t = − 4.407, *P* = 0.000 < 0.001, Fig. 7b; t = − 3.266, *P* = 0.002 < 0.01, Fig. 7c). When the manually calculated ECD was higher than 1500 cells/mm^2^, there was no significant difference between the two systems (t = − 0.462, *P* = 0.646 > 0.05, Fig. 7d; t = − 1.140, *P* = 0.261 > 0.05, Fig. 7e; t = 1.890, *P* = 0.091 > 0.05, Fig. 7f). In Fig. 8, six examples of abnormal images were recognized by the proposed system and *system_normal*. As we can see, the proposed system obviously recognized more cells and had more accurate segmentation results than *system_normal*, further indicating the superiority of the proposed system.

The average ECD variability using the proposed system for 1974 images [12] for 169 eyes in the normal CEC testing set was 0.0387; for the abnormal CEC testing set, we also sampled several LASER IVCM images taken from different locations for each eye to collect a total of 750 images for the 119 eyes (including the original 211 images). The average ECD variability was 0.0962.

**Fig. 9:**
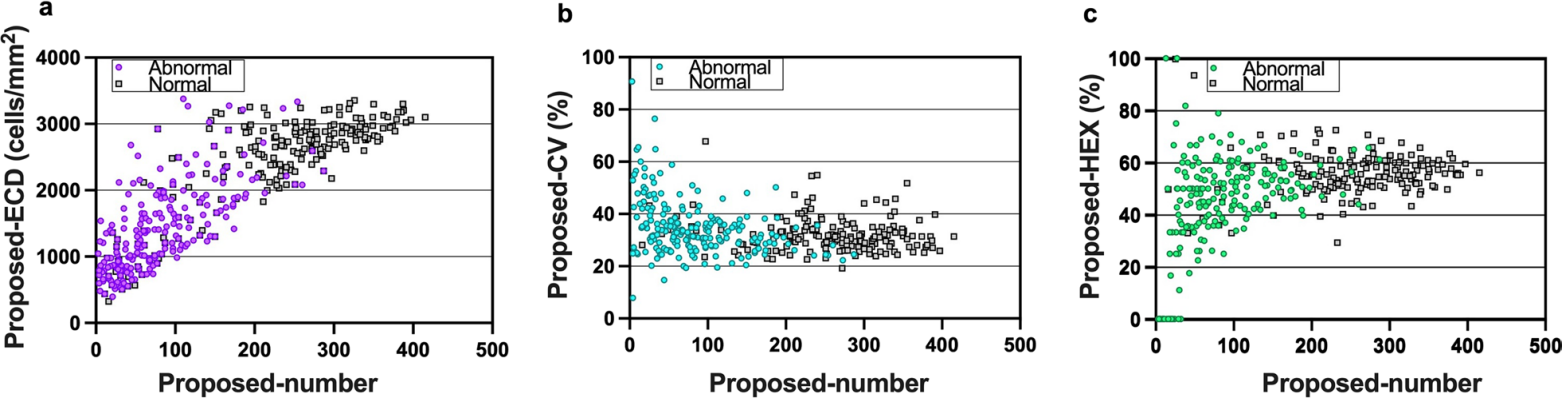
Estimations of ECD, CV and HEX using the proposed system for abnormal (colored circle) and normal (gray square) CEC testing sets, displayed as a function of the number of detected cells in each image. CEC, corneal endothelial cell; ECD, endothelial cell density; CV, coefficient of variation in cell area; HEX, percentage of hexagonal cells

Using the proposed system, the automatedly estimated morphometric parameters were 257 cells, 2648 ± 511 cells/mm^2^, 32.18 ± 6.70% and 56.23 ± 8.69% for the average number of cells, ECD, CV, and HEX, respectively, for the normal CEC testing set and 83 cells, 1450 ± 656 cells/mm^2^, 34.87 ± 10.53% and 42.55 ± 20.64% for the average number of cells, ECD, CV, and HEX, respectively, for the abnormal CEC testing set. As the number of detected cells in the images increased, the ECD from the proposed system increased linearly (Fig. 9a), the proposed CV decreased then approached values between 20% and 40% (Fig. 9b), while the proposed HEX varied considerably at the beginning and approached approximately 50% (Fig. 9c).

Furthermore, the proposed system was very efficient and processed (segmented and quantified) a single image in less than 1 s when run on a 12 GB NVIDIA Tesla K80 GPU and in less than 3 s on a 3.20 GHz Core i7-8700 CPU with 16 GB of RAM.

## Discussion

We developed a robust, accurate, fully automated method for estimating the ECD and morphometric parameters from LASER IVCM images in patients with varying severities of abnormal CECs. This is also the first deep learning system to evalute abnormal LASER IVCM images.

For the abnormal CEC testing set, the Pearson’s correlation coefficient between the automatically and manually calculated ECDs was improved to 0.9447 using the proposed system from 0.9220 with *system_norma*l, and the concordance correlation coefficient was improved to 0.93 (0.91 with *system_normal*). The proposed system thus clearly outperformed *system_normal* in the abnormal CEC testing set. Additionally, when the manually calculated ECD was under 1500 cells/mm^2^, there was a significant difference between the proposed system and *system_normal*. This phenomenon shows that the proposed system achieved an improved capability in recognizing cells in abnormal CEC images. In summary, the proposed system shows significantly improved performance in the abnormal testing set and perfectly complements the deficiencies of our previous system. This large benefit could help the automated system be applied in actual routine clinical work and extend the range of applications for LASER IVCM images.

In Fig. 6, the relative errors decreased as ECD increased in each image. The reason for this might be that the manual counting process only involve the selection of a small ROI on LASER IVCM images for counting cells and calculating the ECD, and there was significant overestimation if the cells were counted in frames smaller than 25% of the image [14]. The ROI could significantly affect the accuracy of the ECD results; thus, it was very necessary to develop a deep learning recognition system for both abnormal and normal CEC images.

Previously, U-Net had shown promising results in cell segmentation via a delineation of the cell borders. Fabijańska demonstrated further proof via the high performance and accuracy of a U-Net-based learning approach for normal specular corneal endothelial images [15]. Currently, CNN technology is used to assess the corneal endothelium from specular microscopy images post-corneal transplant [11, 12]. Those studies mainly focused on ECD, HEX and CV for both healthy and diseased corneas, and demonstrated improved estimation of HEX and CV over previous traditional methods [11, 12]. However, one main limitation that prevents them from being used in the clinic is that they were mainly tested with high-quality images and/or healthy corneas. Although the researchers emphasized the challenging cases involved in their studies, we found that the morphology of the CECs was still normal with respect to that in the images in our study. Through the assessment of the effectiveness of the proposed system, we also showed that CNNs can be used in the morphological recognition and analysis of abnormal CECs in LASER IVCM images. Furthermore, the time of identification was less than 1 s with the use of our deep learning system.

## Limitations

Our system should be further confirmed under cross-validation experimental configuration. In future studies, images should be obtained for both monocentric and multicenter validation of our system. Furthermore, the estimations of morphometric parameters obtained using the proposed system should be tested with large-scale clinical trials.

## Conclusions

This is the first report to count and analyze the morphology of abnormal CECs in LASER IVCM images using deep learning. We demonstrated that CNNs can be used to recognize LASER IVCM images with high noise levels and low quality. Artificial intelligence-based recognition can produce more objective evaluation indicators for LASER IVCM corneal endothelium images and greatly expands the range of applications of LASER IVCM in the field of ophthalmologic operations, such as post-DSAEK.

## Data Availability

The datasets used or analyzed during the current study are available from the corresponding author on reasonable request.
